# Sudden-onset gastrointestinal bleeding in a young adult: diagnostic and therapeutic challenges of a Dieulafoy’s lesion in the jejunum

**DOI:** 10.1186/s40792-024-02064-9

**Published:** 2024-11-22

**Authors:** Shikhar Tripathi, Rakesh Narayanagowda, Sri Aurobindo Prasad Das, Sunila Jain, Samiran Nundy

**Affiliations:** 1 Institute of Surgical Gastroenterology, GI & HPB Oncosurgery and Liver Transplant, Sir Ganga Ram Hospital, New Delhi, India; 2 Department of Pathology (Histopathology Division), Sir Ganga Ram Hospital, New Delhi, India

**Keywords:** Dieulafoy's lesion, Jejunal Dieulafoy's lesion, GI bleeding

## Abstract

**Background:**

A Dieulafoy’s lesion in the jejunum is at an uncommon site but may be the cause of massive gastrointestinal bleeding. It is characterized by a large, tortuous submucosal artery that erodes the overlying epithelium and presents diagnostic and therapeutic challenges due to its atypical location and presentation.

**Case:**

A 30-year-old male presented with sudden onset syncope and the passage of 200–300 ml of red blood-mixed stool. With no major comorbidities, he had hypotension with a blood pressure of 80/50 mmHg, necessitating immediate transfusion of three units of packed red blood cells (PRBCs). Initial endoscopic evaluations, including an UGI endoscopy and colonoscopy, failed to locate the bleeding source. CT angiography identified an active bleed from the first jejunal branch leading to coil embolization. Persistent symptoms prompted capsule endoscopy, revealing angioectasia in the proximal jejunum. Despite haemoclip application and a total of 11 units of blood transfused, his symptoms persisted. He then underwent laparoscopic resection of the jejunal segment containing the polyp, followed by extracorporeal jejuno-jejunal anastomosis. Histopathology confirmed a benign polyp with central ulceration, consistent with a Dieulafoy’s lesion.

**Conclusions:**

Advanced diagnostic techniques like CT angiography and capsule endoscopy played a pivotal role in localizing the bleeding source. Surgical intervention proved curative when less invasive methods failed. The patient’s postoperative course was uneventful, highlighting the efficacy of a multidisciplinary approach. A high index of suspicion and a multidisciplinary approach are essential for successful outcomes.

## Introduction

A jejunal Dieulafoy’s lesion (JDL) represents an exceedingly rare yet significant cause of massive gastrointestinal bleeding. This lesion, first described by the French surgeon Paul Georges Dieulafoy in 1898, typically involves the stomach. However, the jejunum can also be affected, though it remains an uncommon site [1]. This vascular anomaly is characterized by an abnormally large submucosal artery that can cause severe bleeding without an associated ulcer or erosion [2].

The incidence of JDL is not well-documented due to its rarity, but it poses a diagnostic challenge due to its atypical location and nonspecific presentation. Patients often present with severe, life-threatening gastrointestinal haemorrhage, which necessitates prompt diagnosis and management. Advances in diagnostic techniques, including endoscopy and imaging, have improved the identification and treatment of this condition, but its rarity means that clinical experience remains limited [3].

In this case report, we present a patient with a Jejunal Dieulafoy’s Lesion, detailing the clinical presentation, diagnostic workup, and management. This report aims to contribute to the existing literature by providing additional insights into the diagnosis and treatment of this rare but potentially fatal condition, emphasizing the need for awareness among clinicians to improve patient outcomes.

## Case discussion

A 30-year-old male presented with sudden onset syncope and passage of approximately 200–300 ml of red blood-mixed stool. He had profound hypotension (80/50 mmHg) on admission, necessitating the transfusion of three units of packed red blood cells (PRBCs). He had no history of comorbidities.

**Fig. 1:**
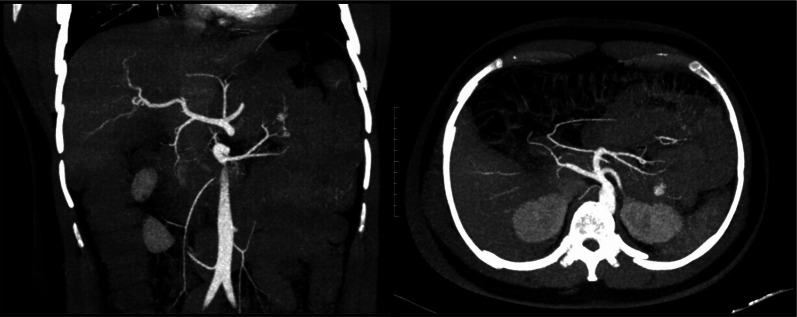
CT Angiogram showing an active bleed from the first jejunal branch

**Fig. 2:**
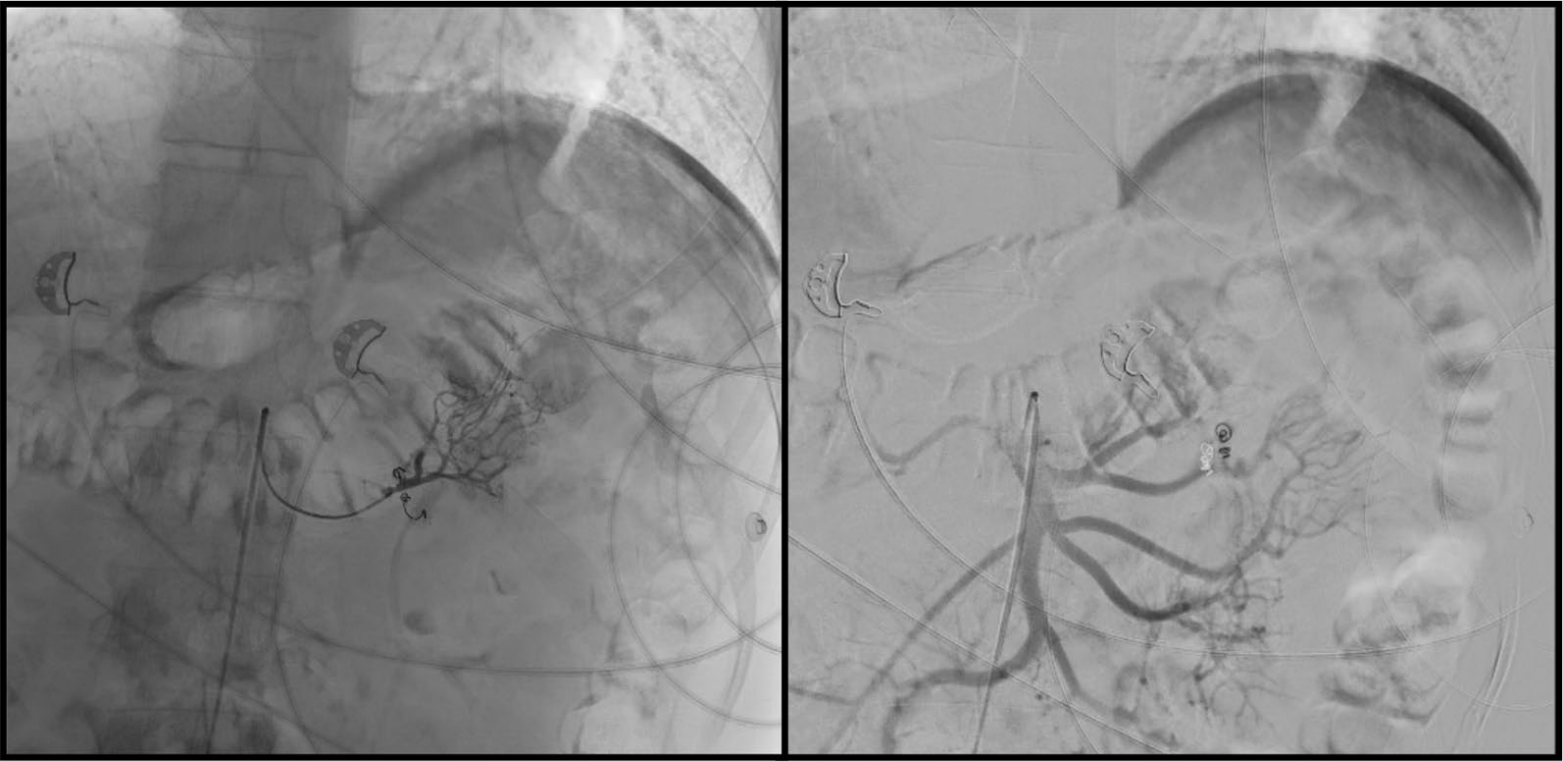
Coil embolization as viewed on a digital subtraction angiogram

At the time of initial presentation, the differential diagnosis for massive gastrointestinal bleeding was broad, given the sudden onset of hematochezia and hemodynamic instability. Potential diagnoses included angioectasia, small bowel malignancy, arteriovenous malformation, and Meckel's diverticulum, all of which can present with severe lower gastrointestinal bleeding. Initial upper GI endoscopy, revealed a normal oesophagus, stomach, and duodenum, and colonoscopy findings were normal for the rectum, sigmoid, transverse, ascending colon, caecum, and terminal ileum. CT angiography subsequently identified an active bleed from the first jejunal branch (Fig. 1), leading to digital subtraction angiography (DSA) and coiling of the affected vessel. Despite these interventions, the patient's symptoms persisted, prompting a second DSA which initially showed no further bleeding. However, ongoing clinical suspicion warranted additional investigation, revealing active hemorrhage from a branch of the pancreatico-duodenal artery, necessitating additional coil embolization (Fig. 2). Persisting symptoms led to capsule endoscopy, which identified angioectasia in the proximal jejunum. Double balloon enteroscopy identified a vascular polyp with active bleeding located 15 cm from the DJ flexure, with haemoclip application performed for tattooing, concluding with the diagnosis of a jejunal polyp. Despite extensive interventions and a total of 11 units of blood transfused, the patient’s symptoms persisted.

**Fig. 3:**
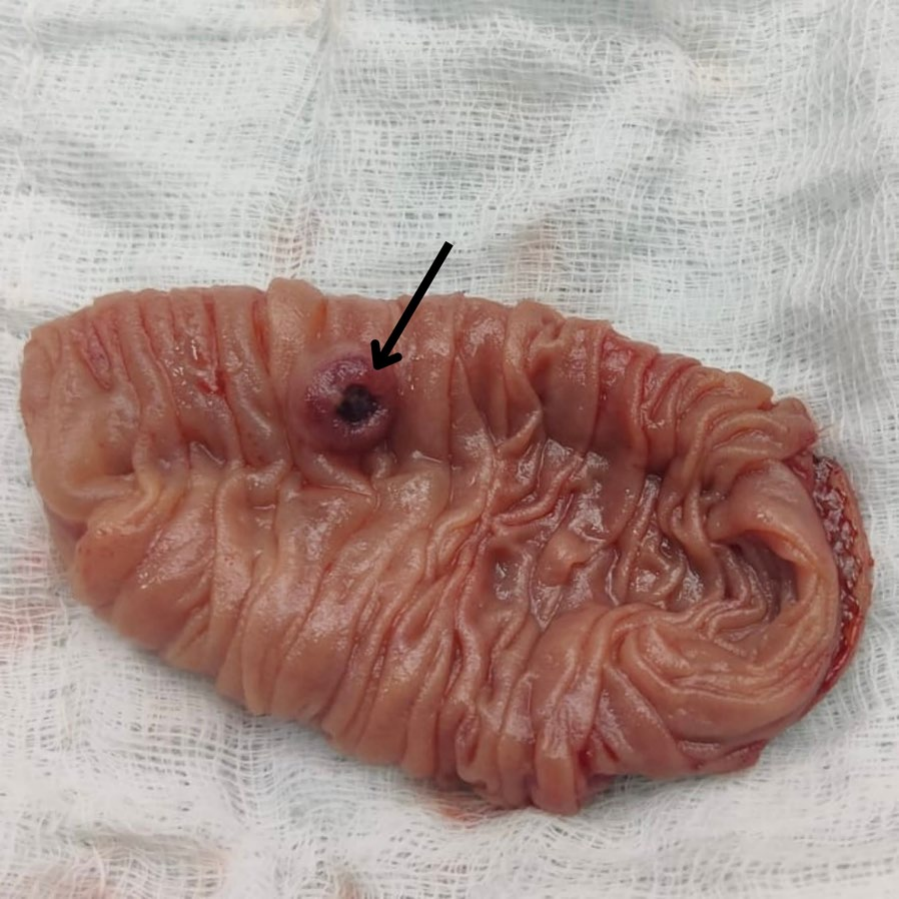
Resected segment, with the polyp (arrow)

**Fig. 4:**
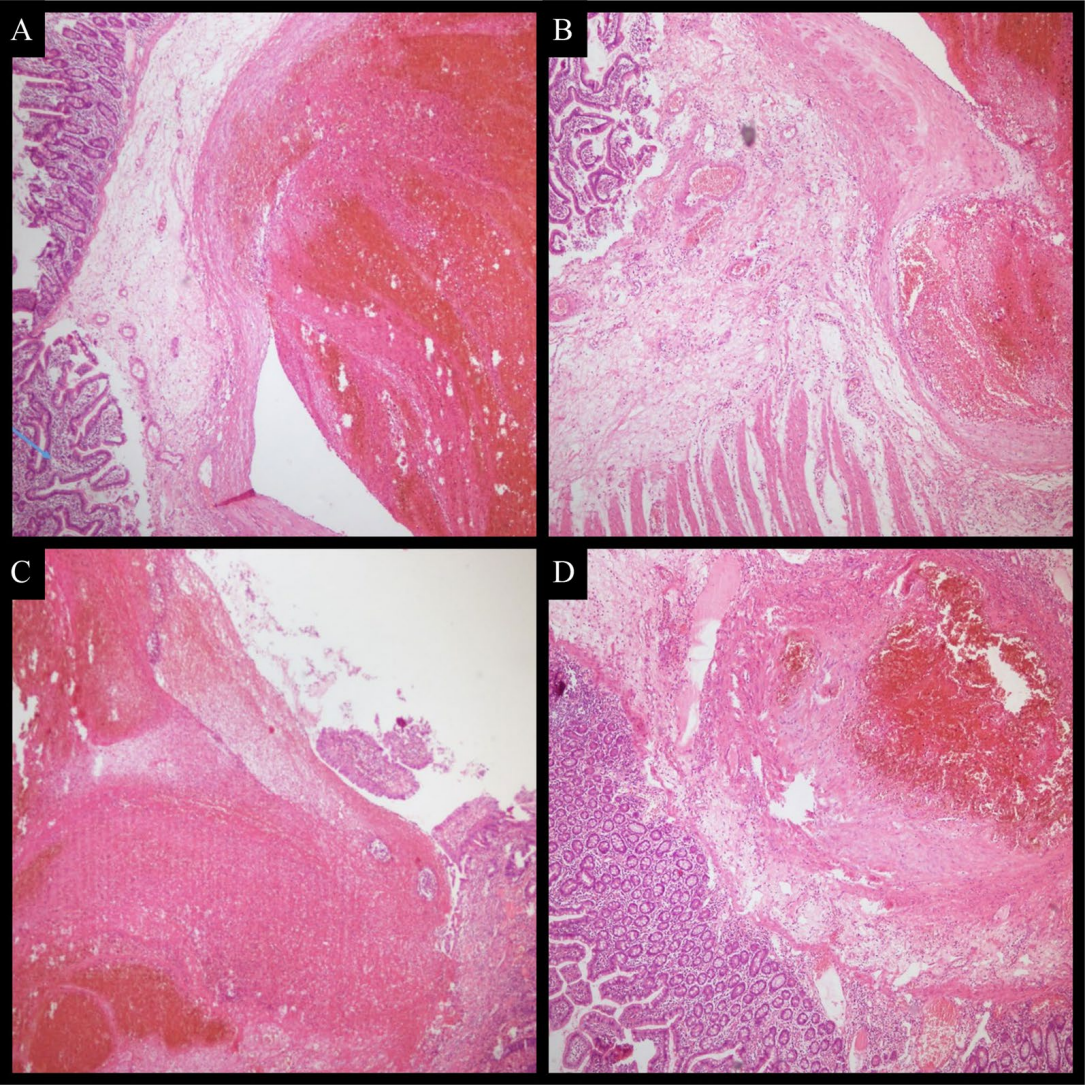
**a** Photomicrograph showing a dilated large calibre submucosal vessel.Overlying normal small intestinal mucosa is seen on left (arrow). Lumen shows an organizing thrombus. (H&E × 20). **b** Vessel having thick wall which is unremarkable. It is reaching the muscularis propria. (H&E × 20). **c** Overlying mucosa is focally ulcerated (H&E × 20). **d** Submucosal vessel with normal overlying small intestinal mucosa (H&E × 20).

On the third day after admission, the patient underwent laparoscopic resection of the jejunal segment harboring the polyp, followed by extracorporeal jejuno-jejunal anastomosis, owing to the rest of the small bowel being healthy and unremarkable on intra-operative analysis. Intra-operative findings included a 0.5 × 0.5 cm polyp situated on the antimesenteric border of the proximal jejunum, approximately one foot from the DJ flexure (Fig. 3). Surgical resection encompassed approximately 10 cm of the jejunal segment. Histopathological examination revealed fragments of small intestinal mucosa with focal ulceration. The submucosa exhibited a large tortuous vessel with a wide diameter protruding into the mucosa with overlying ulceration, filled with haemorrhagic fibrinous material (Fig. 4). Both resected ends were normal, with no evidence of dysplasia, neoplasm, granuloma, or microorganisms, confirming a vascular malformation consistent with Dieulafoy's lesion. The patient's postoperative course was uneventful. He was discharged on post-operative day 7 in a stable condition.

## Discussion

A Dieulafoy's lesion in the jejunum is a rare but may be a cause of major gastrointestinal bleeding. It is characterized by a large, tortuous submucosal artery that erodes the overlying epithelium without an underlying ulcer. The diagnosis and management of a Jejunal Dieulafoy’s Lesion (JDL) present unique challenges due to its rarity, atypical presentation, and the potential for severe, life-threatening haemorrhage.

Our patient, a 30-year-old male without significant comorbidities, presented with sudden onset syncope and significant haematochezia, leading to hypovolaemic shock. This clinical presentation is consistent with other reported cases of JDL, where patients often present with acute gastrointestinal bleeding severe enough to cause haemodynamic instability [1, 2].

Initial diagnostic workup for our patient included upper gastrointestinal endoscopy and colonoscopy, which yielded normal findings up to the proximal jejunum and revealed melaena in the transverse colon. This necessitated further imaging with CT angiography, which identified an active bleed from the first jejunal branch, a finding corroborated by digital subtraction angiography (DSA). Despite initial angioembolization, the bleeding persisted and required further embolization. This diagnostic sequence is reflective of the complexity often encountered in JDL cases, where initial endoscopic evaluations may fail to locate the bleeding source [4]. In this patient, the active hemorrhage from the pancreatico-duodenal artery and the angioectasia in the proximal jejunum represent additional vascular anomalies that may have influenced the severity of bleeding. While the JDL is recognized as the primary source of massive hemorrhage due to its large, tortuous submucosal artery prone to rupture, the concurrent pancreatico-duodenal artery bleed exacerbated the acute blood loss. This interaction between the JDL and other vascular lesions underscores the complexity of the hemorrhagic presentation, necessitating a thorough diagnostic strategy to fully understand and manage the multifaceted sources of gastrointestinal bleeding.

Capsule endoscopy played a crucial role in the diagnosis of our case, revealing angioectasia in the proximal jejunum. This modality has been highlighted in the literature as particularly useful for diagnosing small bowel lesions that are not detectable by conventional endoscopy. Wang et al. demonstrated the utility of advanced imaging techniques, including CT and capsule endoscopy, in diagnosing JDL, particularly in the presence of non-specific clinical presentations [3]. The combination of imaging modalities helped to narrow down the diagnosis, demonstrating how CT angiography and DSA not only identify active bleeding sites but also confirm the presence of vascular anomalies, such as JDL. This approach showcases the critical role of advanced imaging in the diagnostic process, helping clinicians refine differential diagnoses based on findings from sequential investigations.

The management of these lesions requires a multidisciplinary approach. Initial attempts to control bleeding in our patient involved endoscopic techniques and angiographic interventions, including DSA-guided coiling. However, the persistence of bleeding and the patient's ongoing haemodynamic instability necessitated surgical intervention. This aligns with findings by Malik et al. who emphasize the necessity of surgical resection in cases where endoscopic and radiologic measures fail to achieve haemostasis [5].

Our patient underwent laparoscopic resection of the jejunal segment containing the polyp, followed by extracorporeal jejuno-jejunal anastomosis. This surgical approach is consistent with recommendations for managing refractory cases of JDL. The intraoperative findings of a 0.5 × 0.5 cm polyp on the antimesenteric border of the proximal jejunum with central ulceration were characteristic of a Dieulafoy's lesion, as confirmed by histopathological examination. The literature supports surgical resection as a definitive treatment, particularly in cases with recurrent bleeding or failed endoscopic management [2, 6].

Comparatively, the outcomes reported in the literature for surgically managed JDL cases are generally favorable, with the key to successful outcomes lying in timely intervention and the appropriate selection of surgical candidates, particularly when less invasive measures fail [2, 5].

The comprehensive review of literature underscores the variability in clinical presentation, diagnostic approaches, and management strategies for JDL. Kalantari et al. and Oladunjoye et al. emphasize the importance of considering JDL in the differential diagnosis of obscure gastrointestinal bleeding, particularly in cases unresponsive to conventional diagnostic methods [1, 6]. The role of advanced diagnostic tools, such as capsule endoscopy and CT angiography, is highlighted across multiple studies [3, 4].

The underlying pathophysiology of JDL involves a large, tortuous artery in the submucosa, which can cause significant bleeding even without an ulcer or erosion. This vascular malformation is prone to rupture, leading to severe hemorrhage. The exact mechanism leading to the formation of Dieulafoy's lesion is not well understood, but several hypotheses have been proposed, each with merit of their own: Some researchers suggest that Dieulafoy's lesion may arise from a congenital defect in the vascular architecture, resulting in an aberrant artery that fails to taper as it penetrates the submucosa [7]. This persistent large artery can easily erode through the mucosal surface, especially in the jejunum where the mucosal layer is thinner compared to other parts of the gastrointestinal tract. Another hypothesis posits that Dieulafoy's lesions may develop due to acquired vascular changes. Chronic inflammation or repeated minor trauma to the intestinal mucosa might induce local vascular proliferation and the formation of a large, tortuous artery [4]. This is supported by findings of inflammatory markers in the vicinity of the lesion in some histopathological examinations, however this was not exhibited in our case. Howbeit, our hypothesis aligns with the theory of haemodynamic stress leading to remodelling [8, 9]. The high-pressure arterial blood flow in the jejunal arteries, combined with the peristaltic movements of the intestine, could exert mechanical stress on the vascular structures leading to their dilation and tortuosity, leading to the formation of these lesions. Future research should focus on elucidating the molecular and genetic factors underlying the formation of Dieulafoy’s lesions to enhance early diagnosis and improve treatment outcomes.

Management strategies for Jejunal Dieulafoy’s Lesion (JDL) vary significantly depending on the clinical scenario and the patient's response to initial treatments. Endoscopic techniques are typically the first-line interventions due to their minimally invasive nature and effectiveness in controlling bleeding [10]. Techniques such as haemoclip application and tattooing, as demonstrated in our case, are widely used to achieve haemostasis and mark the lesion for potential future interventions. These methods are supported by extensive literature, indicating their efficacy in many cases of gastrointestinal bleeding. However, when bleeding persists despite endoscopic measures, more aggressive interventions are necessary. Surgical intervention becomes crucial in refractory cases [5]. This involves removing the affected segment of the jejunum and performing anastomosis, which not only addresses the immediate bleeding but also reduces the risk of recurrence. The decision to proceed with surgery is often based on the severity of the bleeding, the patient's hemodynamic stability, and the failure of endoscopic and angiographic interventions [11].

The prognosis for patients with Jejunal Dieulafoy's Lesion (JDL) largely hinges on the timeliness of diagnosis and the effectiveness of the management strategies employed. Early identification of the lesion, facilitated by advances in diagnostic techniques such as capsule endoscopy and CT angiography, plays a critical role in improving outcomes [12]. Endoscopic interventions, when successful, provide a minimally invasive solution to control bleeding. However, in cases where endoscopic methods fail, prompt surgical intervention ensures definitive treatment, minimizing the risk of rebleeding and associated complications [13]. The coordinated efforts of gastroenterologists, radiologists, and surgeons are crucial in managing complex cases of JDL, leading to a significant improvement in patient outcomes.

## Conclusion

This case of a 30-year-old male with a Jejunal Dieulafoy's lesion highlights the challenges in diagnosing and managing gastrointestinal bleeding. Despite initial interventions, persistent bleeding necessitated a multidisciplinary approach and surgical resection. Advanced diagnostic techniques like CT angiography and capsule endoscopy were crucial. The successful outcome underscores the importance of prompt diagnosis and comprehensive management. This case reinforces the need for high suspicion and a team-based approach for effective treatment of this rare condition, ultimately improving patient outcomes.

## Data Availability

Not applicable.

## Abbreviations

JDL: Jejunal Dieulafoy’s Lesion
PRBCs: Packed red blood cells
DSA: Digital subtraction angiography
